# Intensive Dialectical Behavior Treatment for Individuals With Borderline Personality Disorder With and Without Substance Use Disorders

**DOI:** 10.3389/fpsyg.2021.629842

**Published:** 2021-08-23

**Authors:** Frank D. Buono, Kaitlyn Larkin, David Rowe, M. Mercedes Perez-Rodriguez, Matthew E. Sprong, Amir Garakani

**Affiliations:** ^1^Yale School of Medicine, New Haven, CT, United States; ^2^Silver Hill Hospital, New Canaan, CT, United States; ^3^Icahn School of Medicine at Mount Sinai, New York, NY, United States; ^4^Department of Social Work and Counseling, Lock Haven University, Lock Haven, PA, United States

**Keywords:** borderline personality disorder, substance usage disorders, dialectical behavior therapy, generalized anxiety, depression

## Abstract

Treatment of borderline personality disorder (BPD) with comorbid substance use disorder can be challenging due to symptom overlap and limited assessment methods. Preliminary evidence has shown promising effectiveness of dialectical behavioral therapy (DBT) for BPD with comorbid substance use disorders. The current study compared the benefits of a 28-day transitional DBT treatment program for individuals with BPD with and without substance use disorders through evaluating the changes in coping skills, generalized anxiety, and depression symptom scales at admission and discharge. A total of 76 patients were split into two groups: Group 1 consisted of individuals with BPD without substance use disorders (*n* = 41), and Group 2 involved individuals with BPD and a substance use disorder (SUD) (*n* = 35). A univariate general linear model showed significant differences between the two groups in improvement of coping skills and depressive symptoms. After a 28-day transitional DBT treatment program there were significant decreases from severe to moderate depression scores in both groups. Our findings support the effectiveness of DBT treatment in patients with comorbid BPD and SUD.

## Introduction

Substance use disorders (SUD) are highly prevalent among individuals with borderline personality disorder (BPD) with 78% of individuals meeting diagnostic criteria (American Psychiatric Association, 2013). BPD and SUD share similar underlying traits such as impaired impulse control and affective dysregulation (Sher and Trull, 2002). Diagnosing and assessing comorbid BPD and SUD can be challenging due to symptom overlap and limited assessment methods (Langas et al., 2012). As separate diagnoses, BPD and SUD are associated with significant impairment (Ramey and Regier, 2018; Marchand et al., 2019; Videler et al., 2019), and their co-occurrence can lead to greater distress and impairment in social and occupational areas of functioning (e.g., less social support, suicide attempts, interpersonal issues, conflicts with legal authorities) (Trull, 2000; Trull et al., 2018). These difficulties are associated with an increased likelihood of relapse and poor treatment outcomes (Grant et al., 2004; Langas et al., 2012; Carra et al., 2015), highlighting the need for effective treatment for individuals with BPD and SUD.

While studies have explored treatment approaches to BPD (Bateman et al., 2015) and SUD (Marchand et al., 2019) individually, there is limited research that evaluates treatment methodologies for co-occurring BPD and SUD. In fact, patients with comorbid SUD are often excluded from clinical trials. A recent systematic review by Lee et al. (2015) examined effective treatments for comorbid BPD and SUD, resulting in three available treatment strategies: Dialectical Behavior Therapy (DBT), a specific, skills-based form of Cognitive Behavioral Therapy (CBT) that promotes acceptance and change, Dynamic Deconstructive Psychotherapy (DDT), a manual-based, modified form of Psychotherapy, and Dual-Focused Schema Therapy (DFST), which combines relapse prevention for substance dependence and work on early maladaptive schemas. Lee et al. (2015) concluded that, despite the absence of a strong evidence base, DBT, and dynamic deconstructive psychotherapy (DDP) showed benefit in reducing symptoms, naming DBT “the first choice” of treatment for individuals with comorbid BPD and SUD.

DBT is an empirically-supported treatment, utilizing skills-based behavioral exercises, that has been effective in increasing distress tolerance, emotional regulation, and improving interpersonal effectiveness (Bloom et al., 2012). DBT was initially designed to treat suicidal patients and successfully utilized as an intensive outpatient treatment program for individuals with BPD (Linehan and Kehrer, 1993). DBT has also been successful in treating various other psychological disorders such as anxiety, mood, and eating disorders (Neacsiu et al., 2010; Webb et al., 2016; Conrad et al., 2017; Linardon et al., 2017). There is, however, only preliminary evidence on the efficacy of DBT for SUD (Stotts and Northrup, 2015) and even fewer trials of DBT for BPD and SUD (Lee et al., 2015).

The purpose of this pilot study was to examine the benefits of a 28-day, transitional, DBT treatment program for individuals with BPD and SUD compared to those with BPD and no SUD through evaluating the changes in DBT-Ways of Coping Checklist (DBT-WCCL) skill use and anxiety and depression symptom scales at admission and discharge.

## Methods

### Participants

Participants were recruited, consented, and completed all intake assessments within 72 h of being admitted to the transitional living program by the first author. The current study was approved by the Yale University Human Subjects Committee. Participants were included in the study if they met the following criteria: (1) Admitted to a 28-day transitional living program within a private psychiatric hospital, (2) willing to complete additional research assessments, and; (3) confirmed a mental status sufficiency (e.g., absence of psychosis, intellectual limitations, dementia) understanding through psychiatric interviews by admission and transitional living medical providers. Individuals were recruited from August 2018 through May 2019.

A total of 94 individuals attempted to enroll in the study at admission. Of the 94 individuals, 18 individuals were prematurely discharged (*n* = 14) or needed a higher level of care (*n* = 4), therefore discharge data was not collected and participants were removed from the analysis. Participants were divided *post-hoc* after completion of treatment into two groups based on DSM-5 criteria for borderline personality disorder and an active substance use disorder (e.g., not in early or sustained remission) and including any substance use disorder (e.g., cannabis, cocaine, opioid, alcohol) except a primary tobacco use disorder (American Psychiatric Association, 2013). Group 1 consisted of individuals with borderline personality disorder without substance use disorders (BPD; *n* = 41), and Group 2 involved individuals with borderline personality disorder and substance use disorder (BPD and SUD; *n* = 35). Diagnoses of patients were made or confirmed at admission by a board-certified psychiatrist. It should be noted that individuals when needed who had SUD were admitted after completing a detox program, to avoid any confounding effects of substance withdrawal.

### Setting

The program is a 28-day, self-pay transitional living program (TLP) within a private psychiatric hospital. The TLP consists of several separate houses focusing on treating patients with either dual diagnoses, psychoses, or mood and personality disorders. Patients are either referred from inpatient psychiatric or medical hospitals or by their outpatient providers or can self-present from home without a referral. The TLP that provides DBT admits eligible patients with a range of potential diagnoses and co-morbidities; however, there is a special focus on individuals with personality disorders. Although patients do live on the hospital campus for their 28 days while doing the DBT TLP, they are not technically on a locked inpatient unit and thus need to be without acute suicidal thinking, intent or plan, and stable enough to participate in treatment in a transitional setting. Psychosocial treatments integrated DBT specifically tailored to personality disorders in both group-based treatment and individual sessions. The multi-disciplinary team (i.e., nurses, social workers, psychiatrists, and counselors) received training through Behavioral Tech, a comprehensive, DBT training institute, and received on-site supervision every 2–3 months from a Behavioral Tech consultant. Ancillary therapies were incorporated into treatment (e.g., yoga, music therapy, art therapy). Patients received, at minimum, five 1-h DBT group sessions and two individual 1-h DBT coaching sessions per week for 4-weeks. All sessions were built off empirically-based DBT protocols, DBT Skills Training Handouts and Worksheets (Linehan, 2014).

All patients in the program received the same schedule of DBT groups and all staff (i.e., therapists, psychiatrists, residential counselors) attended a weekly process group and regular supervision from outside consultants. There was no substance use specific group therapy offered in the DBT program, but patients with substance use disorders were offered the option to attend daily AA/NA meetings. Also, there likely were between-subject differences in the content of individual supportive therapy received, as patients were not all assigned the same therapist or psychiatrist.

### Treatment Outcome Assessments

The **Generalized Anxiety Disorder−7 (GAD-7)** is a 7-item, self-report assessment that measures symptoms of generalized anxiety disorder over the past 2 weeks on a 4-point scale (*1* = *not at all, 4* = *nearly every day*) (Spitzer et al., 2006). Reliability of this assessment demonstrated a Cronbach's alpha of (α = 0.92) (Spitzer et al., 2006). The utilization of the GAD-7 assessment was based on recent research indicating improvement of GAD when BPD symptoms are addressed (Keuroghlian et al., 2015). The **Patient Health Questionnaire-−9 (PHQ-9)** is a 9-item, self-report assessment that evaluates the nine diagnostic criteria for major depressive disorder (MDD) over the past 2 weeks on a 4-point scale (*1* = *not at all, 4* = *to a great degree*) (Kroenke et al., 2001). Reliability of this assessment demonstrated a Cronbach's alpha of 0.89 (α = 0.89) (Kroenke et al., 2001). Given the high co-occurrence of BPD with depressive behavior (Richman and Unoka, 2015), the PHQ-9 was implemented to objectively measure the change in severity of participants' depressive symptoms within treatment. The **DBT-Ways of Coping Checklist (DBT-WCCL)** is a 59-item, self-report assessment that evaluates how individuals cope and manage emotions in stressful situations, both positively and negatively, through utilizing skills learned in DBT sessions. This assessment measures these skills over the past 30 days on a 4-point scale (*0* = *never used, 3* = *regularly used*) (Neacsiu et al., 2010). Three subscales are scored: *skills use, general dysfunctional coping*, and *blaming others*. In the original validation study, DSU (0.92 ≥ α ≥0.96) and DCS (0.87 ≥ α ≥0.92) showed excellent internal consistency (Neacsiu et al., 2010).

### Procedure

All surveys and demographics were completed by the participant through a secure and encrypted Qualtrics (www.qualtrics.com) webpage that was administered on an Apple iPad. Participants were re-evaluated 24-h prior to discharge. Patients were compared in two groups: BPD with a DSM-5 diagnosis of a substance use disorder (BPD and SUD), and BPD without SUD (BPD).

### Data Analysis

*T*-tests and chi-square tests were used to compare participants' demographics and clinical features across the two groups. Univariate general linear models (GLM) with robust variance estimation and autoregressive (AR1) working correlation structure with intercept were used to evaluate group (BPD and SUD vs. BPD) differences in treatment outcomes (anxiety [GAD-7] and depressive [PHQ-9] symptoms and coping skills [DBT-WCCL]) across time (intake, discharge). Multivariate analyses were bypassed due to low scale intercorrelation. Results were considered statistically significant when the probability of a Type I error was <0.05 (*P* < 0.05). For each univariate analysis, the assumption of sphericity was tested using an examination of the Huynh–Feldt (H–F) epsilon for the general model. If this statistic was >0.75, sphericity was considered to have been met, and the unadjusted univariate statistic was used. If epsilon was <0.75, a violation of the assumption of sphericity was considered to have occurred, and the H–F adjusted statistic was used to determine significance and accompanying 95% confidence intervals (CIs) were included.

## Results

A total of 94 individuals were enrolled during the study period. Of the 94 participants, 76 completed the assessments, with the 18 individuals not completing either being prematurely discharged (*n* = 14) or needing a higher level of care (*n* = 4). Group 1 consisted of individuals with BPD without SUD (BPD; *n* = 41), and Group 2 was composed of individuals with BPD and SUD (BPD and SUD; *n* = 35). There were no significant differences between the two groups (BPD and SUD vs. BPD) in any of the sociodemographic or clinical history variables. Patients with BPD and SUD (*n* = 35, M = 33.9, SD = 14.7) and BPD (*n* = 41, M = 33.2, SD = 18) were similar in age. There were slightly more females in the BPD group (*n* = 24) than the BPD and SUD group (*n* = 16). A majority of both groups were single at admission 68% (BPD and SUD), 63% (BPD) and unemployed 43% (BPD and SUD), to 29% (BPD). Interestingly, both groups self-reported high rates of previous trauma events 69% (BPD and SUD) and 54% (BPD). All demographics and clinical history variables can be seen on Table 1.

**Table 1:** Sociodemographic and clinical history of participants.

	**BPD and SUD**	**BPD**	***P-*value**
	**(*n =* 35)**	**(*n =* 41)**	
**Age (SD)**	33.9 (14.7)	33.2 (18)	0.886
**Gender**			
Male	19 (54%)	16 (39%)	
Female	16 (46%)	25 (58%)	
**Marital status**			0.836
Single	24 (68%)	25 (63%)	
Married	7 (20%)	10 (25%)	
Divorced	2 (6%)	4 (10%)	
Separated	2 (6%)	1 (2.5%)	
**Employment status**			0.464
Employed	7 (20%)	10 (25%)	
Homemaker	3 (8%)	1 (2%)	
Student	8 (23%)	12 (29%)	
Retired	1 (3%)	4 (10%)	
Workers comp	1 (3%)	0 (0%)	
Receiving SSI/SSD	0 (0%)	2 (5%)	
Unemployed	15 (43%)	12 (29%)	
**Family history of substance use**	20 (61%)	18 (42%)	0.108
**Previous mental health treatment**	16 (46%)	18 (42%)	0.33
**Family history of mental health issues**	27 (77%)	32 (78%)	0.888
**History of traumatic experiences**	24 (69%)	22 (54%)	0.19
**Duration of substance use history in years (M; SD)**	7.3 (11.1)	-	-
**Primary substance use**			
Marijuana	11 (31%)	–	
Cocaine	5 (14%)	–	
Heroin	16 (46%)	–	
Amphetamines	3 (9%)	-	

*SD, Standard deviation; SSI, social security income; SSD, social security disability; BPD, Borderline Personality Disorder; SUD, Substance Use Disorder*.

Intake and discharge values for the GAD, PHQ, and DBT-WCCL across groups can be seen on Table 2. The univariate GLM examining group differences in the treatment outcome assessments (dependent variables) showed that there was a significant difference between BPD and SUD and BPD for depression scores [95% CI = 18.4–21.3; *F*_(1, 0.85)_ = 5.16, *p* = 0.024], but not for anxiety scores (95% CI = 14.5–17.1; *p* = 0.370). Both groups (BPD and SUD; BPD) demonstrated decreased depression scores after treatment (compared to intake scores); however, the BPD group showed a larger improvement (31% reduction in symptoms from intake to discharge) compared to the BPD and SUD group (29% reduction in symptoms from intake to discharge) when comparing pretreatment to post-treatment. There were no significant differences between the two groups in generalized anxiety symptom change within treatment. The groups significantly differed in decreasing general dysfunctional coping subscale [95% CI = 2.6–2.8; *F*_(1, 2.01)_ = 12.3, *p* < 0.001]. However, there were no significant group differences in the DBT skill use (95% CI = 2.7–3.0; *p* = 0.059) or blaming others (95% CI = 2.0–2.2; *p* = 0.358) subscales. In evaluating within-group differences, significant changes were noted after treatment. All three DBT skills sub-factors changed, characterized by an increase of skill use and a decrease of dysfunction and blaming, in a relatively short period of time. Interestingly, the BPD and SUD group (28.1%) showed a larger improvement in the general dysfunction subscale than the BPD group (22.5%).

**Table 2:** Differences across groups (BPD and SUD vs. BPD alone) in changes in clinical symptoms, coping skills and functioning after treatment.

**Assessments**	**Time period**	**BPD and SUD (M, SD)**	**BPD (M, SD)**	***P*-value**
Anxiety	Intake	18.4 (7.1)	19.6 (6.1)	
	Discharge	12.9 (4.3)	13.9 (5.7)	<0.01
Depression	Intake	24.3 (6.5)	24.1 (6.6)	
	Discharge	17.1 (6.1)	16.8 (7.5)	<0.01
**Coping skills**				
DBT skill use	Intake	2.7 (0.5)	2.5 (0.5)	
	Discharge	3.2 (0.4)	3.0 (0.6)	<0.01
General dysfunctional coping	Intake	3.2 (0.6)	3.1 (0.5)	
	Discharge	2.3 (0.5)	2.4 (0.7)	<0.01
Blaming others	Intake	2.7 (0.9)	2.3 (0.8)	
	Discharge	2.0 (0.4)	1.9 (0.8)	<0.01

*M, Mean; SD, Standard Deviation; DBT, Dialectical behavior therapy*.

*Anxiety: self-reported Generalized Anxiety Disorder Scale−7 (GAD-7); Depression: self-reported Patient Health Questionnaire−9 (PHQ-9); Coping skills: DBT-Ways of Coping Checklist (DBT-WCCL)*.

## Discussion

The current study aimed to evaluate the differences in DBT treatment outcomes between individuals diagnosed with BPD who either do or do not have a comorbid SUD. Leveraging the use of a 28-day transitional program that integrated an intensive DBT protocol, this study compared clinical improvement among patients with BPD to those with BPD with comorbid SUD. The findings in this study raise several important points. Regardless of SUD comorbidity, all BPD patients showed a significant decrease from severe to moderate depression and generalized anxiety scores during a 28-day inpatient or transitional treatment experience. This is noteworthy, given the brief duration of the intervention compared to the usually much longer outpatient DBT treatment (Bloom et al., 2012). Our results suggest that BPD patients with SUD can derive psychiatric symptom benefit from a standard DBT program. However, the supplementation of SUD treatment and carryover effect need to be evaluated in more detail.

A significant finding was that the treatment effects were smaller among those with BPD and SUD for DBT-WCCL general dysfunction scores and depression scores. General dysfunction is a key target in DBT therapy and predictor for individuals with personality disorders. Difficulties establishing and maintaining emotional regulation (e.g., negative affect, acceptance of emotions) (Probst et al., 2019) can be heightened if there is a co-occurring SUD (Grant et al., 2004). The preliminary findings of the current study indicate that while emotional dysfunction significantly decreased in both groups, the improvement was more modest among those with SUD. Surprisingly, pre-post changes in skill use and blaming others were not significantly different between groups. These findings extend the utility of the DBT for treatment of BPD (Cristea et al., 2017) and echo the results of (Trull et al., 2018) that roughly half of the patients with BPD have at least one current SUD diagnosis. Treatment of general dysfunction has been previously reported to increase the likelihood to facilitate change in reducing SUD issues by triggering the disorder components that co-occur within SUD and BPD (e.g., affective instability or impulsivity) (Dimeff and Linehan, 2008).

To the author's knowledge this is the first comparison of the DBT-WCCL scores between those who have BPD with vs. without SUD, thus adding to the current literature on the utility of the measure and effectiveness of the measure as a psychometric tool to assess treatment outcomes. The current findings did not show significant differences between the two groups in terms of skill use, nor differences in blaming others. In evaluating within-group differences, significant changes were noted. Shifts were found in all three sub-factors, characterized by an increase of skill use and a decrease of dysfunction and blaming, in a relatively short period of time. While previous research (Robinson et al., 2018; Cavicchioli et al., 2019) has shown the impact of the DBT-WCCL over the course of a standard outpatient treatment time frame, very few articles have utilized and implemented this assessment in intensive transitional treatment or hospital settings (Stein et al., 2016). While the current study evaluated short-term change (under 30 days), patients were given the opportunity to practice coping skills in real world situations. Therefore, future studies should consider evaluating the sub-factors of the DBT-WCCL in patients with co-occurring SUD and BPD.

### Limitations

Notably, this study did not include post-treatment follow-ups, thus limiting its potential external validity. We can only hypothesize a correlational relationship between the change of the variables. Yet, there were changes in three different psychometric scales. Second, this research cannot interpret if the effects of change were solely due to the change of the environment and/or exposure to treatment. It cannot be certain that DBT treatment caused changes in anxiety, depression, and coping skills. However, given the extensive literature published on DBT (Cristea et al., 2017), and the corresponding training of the clinicians, it is unlikely that the changes occurred spontaneously. Furthermore, if the core concepts of DBT were not addressed in the intensive transitional treatment, then variants of the sub-factors would have an inverse reaction. Another limitation was the expense of the transitional living program. This is a problem generally with DBT, but transitional treatment is often only available to the wealthy or those with generous insurance, raising the question of generalizability. The current facility did offer scholarships to individuals on a case-by-case basis, but the researchers were not privy to the information. Another limitation of the study addresses the concern that in a controlled residential environment that all patients will reduce symptoms by blocking access to the substance or the stressor. While this limitation may be true for some, a part of DBT therapy focuses the client to address the stressor(s), and in fact can increase the anxiety or depressive symptoms temporarily so that patient can learn appropriate skills to combat the stressors. The final limitation of the current study was the large proportion of individuals who dropped out of the study prior to completion. Future studies need to validate the current findings, on a larger randomized control trial.

## Conclusions

In the current study of the effects of a 28-day, intensive, transitional, DBT treatment program, significant changes between admission and discharge were found for individuals diagnosed with BPD with and without co-occurring SUD across multiple measures. The significant reduction of general dysfunction while in an intensive treatment program within the shortened time period requires future investigation; however, it does provide evidence that supports DBT as an empirically based strategy for BPD treatment.

## Data Availability Statement

The raw data supporting the conclusions of this article will be made available by the authors, without undue reservation.

## Ethics Statement

The studies involving human participants were reviewed and approved by Yale University Human Subject Committee. The patients/participants provided their written informed consent to participate in this study.

## Author Contributions

FB designed the study and wrote the methodology and discussion. KL was the research assistant for the study and wrote the introduction. DR provided mentorship, provided edits of the revision, and constructed the tables. MP-R provided mentorship of FB and MS with methodology and results and provided edits of the revision. MS wrote the results and provided edits of the revision. AG was medical professional at the hospital, provided edits of the revisions, and helped FB with medical sections of the methodology. All authors contributed to the article and approved the submitted version.

## Conflict of Interest

The authors declare that the research was conducted in the absence of any commercial or financial relationships that could be construed as a potential conflict of interest.

## Publisher's Note

All claims expressed in this article are solely those of the authors and do not necessarily represent those of their affiliated organizations, or those of the publisher, the editors and the reviewers. Any product that may be evaluated in this article, or claim that may be made by its manufacturer, is not guaranteed or endorsed by the publisher.
